# Magnetic Resonance Imaging Evaluation of Multifidus Muscle in Patients with Low Back Pain after Microlumbar Discectomy Surgery

**DOI:** 10.3390/jcm12196122

**Published:** 2023-09-22

**Authors:** Halil Cihan Kose, Serdar Onur Aydin

**Affiliations:** 1 Department of Pain Medicine, Health Science University Kocaeli City Hospital, 41060 Kocaeli, Turkey; 2 Department of Neurosurgery, Health Science University Dr. Lutfi Kirdar Training and Research Hospital, 34120 Istanbul, Turkey; dr.serdaraydin@gmail.com

**Keywords:** magnetic resonance imaging, multifidus muscle, lumbar spine, minimally invasive spine surgery, microlumbar discectomy, low back pain, cross sectional area

## Abstract

Cross-sectional area (CSA) and signal intensity ratio (SIR) of the multifidus muscle (MFM) on magnetic resonance imaging (MRI) was used to evaluate the extent of injury and atrophy of the MFM in patients with negative treatment outcomes following microlumbar discectomy (MLD). Negative treatment outcome was determined by pain score improvement of <50% compared to baseline. Patients in groups 1, 2, and 3 were evaluated at <4 weeks, 4–24 weeks, and >24 weeks postoperatively, respectively. The associations between the follow-up, surgery time and the changes in the MFM were evaluated. A total of 79 patients were included, with 22, 27, and 30 subjects in groups 1, 2, and 3, respectively. The MFM SIR of the ipsilateral side had significantly decreased in groups 2 (*p* = 0.001) and 3 (*p* < 0.001). The ipsilateral MFM CSA significantly decreased postoperatively in groups 2 (*p* = 0.04) and 3 (*p* = 0.006). The postoperative MRI scans found significant MFM changes on the ipsilateral side in patients with negative treatment outcomes regarding pain intensity following MLD. As the interval to the postoperative MRI scan increased, the changes in CSA of the MFM and change in T2 SIR of the MFM showed a tendency to increase.

## 1. Introduction

Low back pain (LBP) is a major problem worldwide and exerts an enormous personal and socioeconomic burden. It is estimated that up to 80% of individuals will encounter at least one episode of this condition during their lifetime [1]. Lumbar disc herniation (LDH) stands out as a prevalent underlying factor behind a number of cases involving LBP. LDH is defined as the shift of intervertebral disc material beyond the boundaries of the disc [2]. This condition often arises due to various factors, including disc degeneration, vertebral instability, axial overloading, genetic predisposition, and traumatic incidents [3]. The pathophysiology of displaced disc material is a combination of mechanical compression of the nerve by the bulging nucleus pulposus and chemical irritation of the nerve roots by local increase in inflammatory cytokines [4,5].

Generally, once multidisciplinary rehabilitation, pharmacologic therapy, and lifestyle-modifying treatments have failed, surgical management is recommended for severe, persistent cases [6]. One of the most frequently performed surgical procedures for symptomatic LDHs is microlumbar discectomy (MLD), which has been shown to provide excellent short-term clinical outcomes, including early patient recovery and rapid return to work [7]. This approach offers several potential benefits, such as smaller incisions of the skin and fascia, less traumatic surgical procedures, and reduced postoperative pain and hospital stays. However, despite its success, up to 30% of patients may still experience dissatisfaction with the procedure [8,9,10,11,12].

Recent developments in the understanding of the biomechanics of LBP have emphasized the crucial role of muscular stabilization in maintaining the neutral zone range of motion in the lumbar region. The lumbar multifidus muscle (MFM) is an important stabilizer of the neutral zone and plays a crucial role in both rotating and stabilizing the lumbar spine [13,14]. Several studies have shown that the morphology of the MFM on magnetic resonance imaging (MRI) is associated with LBP [15,16]. Alterations in its morphology, including a reduction in the functional cross-sectional area due to increased fat infiltration or a decrease in the total cross-sectional area, could potentially contribute to LBP and decreased functional ability. Due to being solely innervated by a single nerve, the MFM is vulnerable to injury and atrophy after spinal surgery. To date, several studies have provided evidence indicating that alterations in the morphology of the MFM after a surgical procedure are associated with poorer clinical outcomes, although debate is still ongoing regarding this effect [17,18,19,20]. Furthermore, an association between the preoperative morphological state of the MFM and clinical outcomes following surgery in patients with unsatisfactory postoperative results has not been well established.

MRI is considered the optimal imaging modality for evaluating spinal pathology and determining the suitability of surgical management [21]. MRI has also become an increasingly popular method for functional muscle monitoring and provides quality information on soft tissue [22]. Furthermore, the axial MRI scan is a reliable tool for examining the morphology of the MFM, with established grading methods that utilize fat infiltration or total cross-sectional area (CSA) to assess muscle quality [23].

To date, several research studies investigating the association between the preoperative measurements of morphological MFM conditions and negative clinical outcomes after MLD were published [24,25]. However, the association between the changes from preoperative to postoperative morphological MFM condition and persistent LBP after MLD remains incomplete. The aim of this current trial was to investigate the association between morphological changes in the MFM muscle on MRI and negative clinical outcomes after MLD. Additionally, we evaluated the correlation between the interval after surgery and alterations in MFM condition on MRI.

## 2. Materials and Methods

### 2.1. Patient Selection

Patients with a primary complaint of LBP combined with radicular leg pain due to single-level LDH confirmed by MRI were included in this study, all of whom underwent MLD from May 2019 to May 2022 at Health Science University Umraniye Training and Research Hospital. Authors retrospectively searched the patient’s electronic medical history records and image archive system to obtain variables. Inclusion and exclusion criteria were determined based on previous studies and discussions among the authors of this study [26,27]. The inclusion criteria for surgery were as follows: A Visual Analogue Scale (VAS) pain score of 4 that is unresponsive to conservative treatment modalities, including nonsteroidal anti-inflammatory drugs and physical therapy for at least three months. All patients had undergone MLD. MRI evaluation was performed 2 times: preoperatively and postoperatively. The second MRI evaluation was performed for patients with negative outcomes. Negative treatment outcome was determined by VAS score improvement of <50% at the follow-up point compared to baseline. MRI scans were performed on all subjects with negative treatment outcomes to assess the intensity of the MFM and CSA. The exclusion criteria for this study are as follows: spine disorders, including segmental instability, scoliosis, and spondylolisthesis; more than one spinal level requiring surgery; a history of previous back surgery performed before this study; neurological disorders that may affect the central or peripheral nervous system such as polyneuropathy and multiple sclerosis; psychiatric disorder; having pathological conditions such as infection, fracture or tumor; and the presence of other systemic disease. The STROBE checklist was used to help design and conduct this study.

### 2.2. Surgical Procedure

As a routine procedure, a two-to-three-centimeter longitudinal midline incision is marked with a sharp scalpel after intraoperative radiography confirms the target level. Subcutaneous dissection with electrocautery then reveals the lumbar fascia over the midline. The MFM is released subperiosteally from the spinous process on one side out to the facet joints with a Cobb elevator after the muscle aponeurosis is incised just off the midline on the side of the approach. The lamina to be performed laminectomy is revealed, retractors are placed, and the microscope is positioned to the field.

After partial hemilaminectomy, the ligamentum flavum is exposed, released, and excised. The nerve root that has been revealed and its associated epidural fat should be visible after the ligamentum has been retracted or removed using a Kerrison rongeur. Once the nerve root has been located, the herniated or fragmented disc tissue is removed during a disc excision procedure.

After adequate nerve decompression, using absorbable sutures, the lumbar fascia and subcutaneous layers are stitched shut. The skin is then stitched shut in the manner preferred by the surgeon [26,27].

### 2.3. Radiological Evaluation

**Figure 1:**
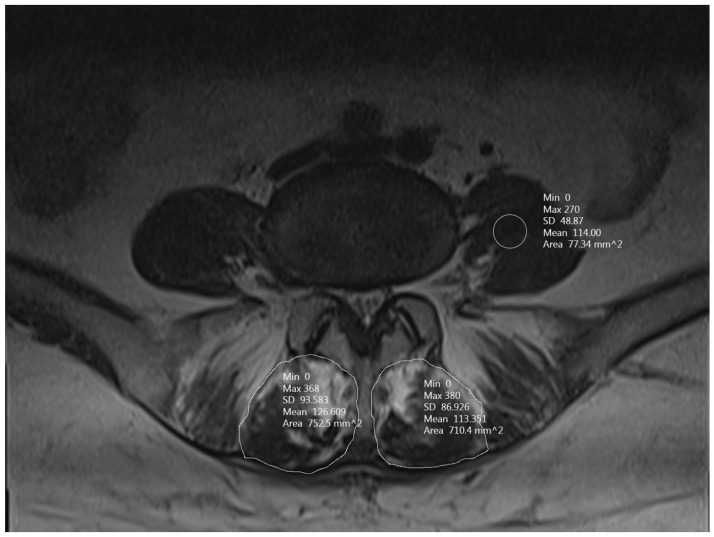
Multifidus muscles (MFMs) on MRI after surgery.

**Figure 2:**
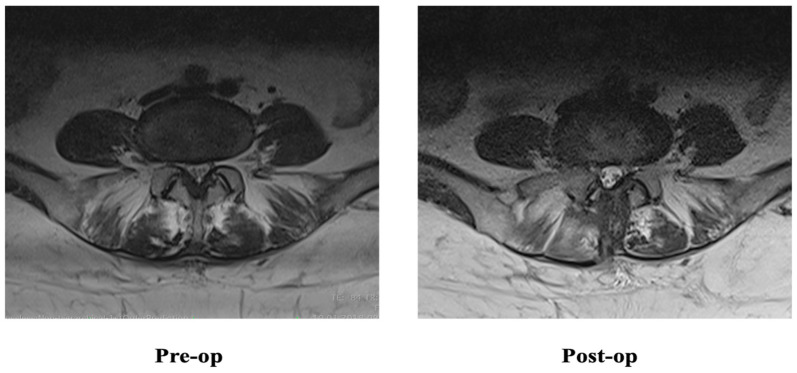
Changes in multifidus muscles on magnetic resonance image after microlumbar discectomy.

MRI scans were performed using a 1.5-Tesla system (Siemens, Munich, Germany) before the operation and at the follow-up point. The interval between the second MRI study and surgery was recorded. The resulting images were analyzed using picture archiving and communication system imaging software (PACS- 3.0.11.4). The calculation of the mean signal intensity ratio (SIR) and CSA of the MFM was performed utilizing T2-weighted MRI scans, employing methodologies established in previously published studies [17,28]. The measurements were performed on both the ipsilateral (surgical) and contralateral sides, with MFM signal intensity quantitatively assessed using the grayscale histogram of the PACS software. To determine the signal intensity, a region of interest (ROI) was delineated, encompassing the entire outer boundary of the muscle, accounting for any intramuscular fat. Concurrently, the psoas muscle’s signal intensity on the corresponding axial scan was assessed by placing a circular ROI with an area of 0.773 cm^2^ precisely at the center of the left-sided psoas (Figure 1). The SIRs of the MFM to the ipsilateral and contralateral psoas muscles, as well as the percentage changes between the baseline and post-operative values, were documented (Figure 2).

Both the SIR of the MFMs to that of the psoas muscle and the CSA of both sides were analyzed and recorded. SIR: signal intensity ratio, CSA: cross sectional area.

### 2.4. Outcome Data and Assessment

The clinical and demographic data of the patients, the 100 mm VAS score on a scale from 0 (no pain) to 100 (worst imaginable pain), the period from surgery to the follow-up MRI evaluation, surgery time, and surgery level were recorded [29]. The data obtained from patients who underwent two MRI scans (before the operation and during the follow-up) were evaluated. The correlations between the time interval until the follow-up assessment and alterations in the MFM, including SIR, CSA, and the percentage of SIR and CSA changes on both sides were evaluated. In addition, we examined whether there is a correlation between the length of operation time and the changes observed in the MFM. Upon completion of data collection, subjects were classified into three groups based on the timing of their follow-up MRI to investigate the correlations between the period from surgery to the follow-up MRI and MFM changes. Patients in groups 1, 2, and 3 were assessed at <4 weeks, 4–24 weeks, and >24 weeks postoperatively, respectively. We analyzed changes in the MFM parameters among these three groups. There were no missing data regarding the demographic, clinical, and radiological data of the included patients in this study.

### 2.5. Statistical Analysis

Statistical analysis was performed with IBM SPSS Statistics version 20. Applying appropriate statistical methods, ensuring complete data, and implementing statistical data analyst blinding methods were utilized to address potential sources of bias. The normal distribution of the data was assessed using the Kolmogorov-Smirnov test. The associations between the follow-up and the changes in MFM were evaluated using Pearson’s correlations. Moreover, the associations between the surgery time and the changes in the MFM were assessed using Pearson’s correlations. One-way analysis of variance for continuous variables and chi-square test or Fisher’s exact test for categorical variables were performed to assess differences between the groups. To compare between groups, we analyzed the nonparametrically distributed data by performing the Kruskal-Wallis test. Furthermore, the Mann-Whitney U test was performed for group comparison as a post hoc test. A *p* value of <0.05 was considered statistically significant.

## 3. Results

### 3.1. Study Population

A total of 79 patients who met the inclusion criteria were included, with 22, 27, and 30 subjects in groups 1, 2, and 3, respectively. The baseline demographic data of the subjects are summarized in Table 1. Among the surgery levels performed in this present study, the most frequent level was L4–L5 (n = 33, 41.7%), followed by L5–S1 (n = 30, 37.9%) (Table 2). Table 3 provides a summary of the surgery data and outcomes. The mean surgery time was 75.11 ± 22.8 min, and the average time interval to the follow-up assessment was 130.41 ± 58.13 days. The VAS score decreased from 7.05 ± 1.18 to 4.59 ± 1.21 at follow-up. No procedure-related complications were reported in any patients during the follow-up period, such as nerve root injury, discitis, dural tears, and great vessel or retroperitoneal injury.

The average time from MLD to the postoperative MRI was 11.64 ± 8.58, 95.16 ± 44.25, and 284.43 ± 84.23 days for groups 1, 2, and 3, respectively. No significant differences were found among the groups in terms of demographic data, surgery level, surgery time, or average VAS score.

### 3.2. Correlation

There was no significant association between the surgery time and MFM changes for both the ipsilateral (r = 0.12) and contralateral (r = 0.18) sides. The period from surgery to the postoperative MRI evaluation was associated significantly with the percentage of alteration in the MFM SIRs (r = 0.47; *p* < 0.001) and MFM CSA for the surgical (r = 0.44; *p* < 0.001) side. However, no significant correlation was found between the period from operation to the follow-up MRI and MFM SIRs changes (r = 0.04) and MFM CSA (r = 0.17) for the contralateral side.

### 3.3. MFM SIR

**Figure 3:**
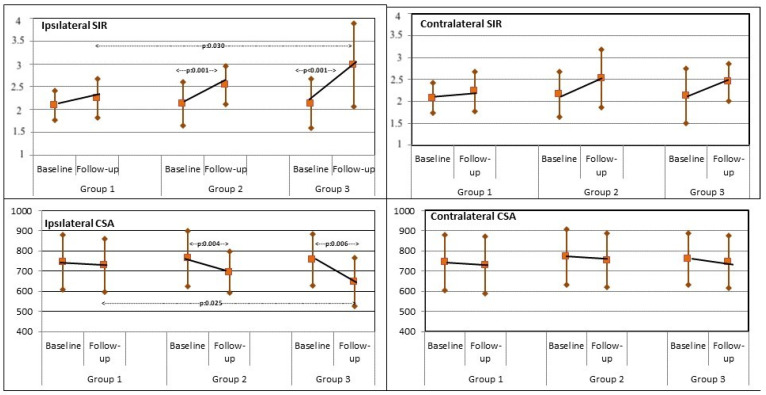
Change between the baseline and follow-up value.

The SIR of the surgical side at the follow-up examination significantly increased compared to the baseline in groups 2 (*p* = 0.001) and 3 (*p* < 0.001). However, no significant difference was apparent in group 1. There was a significant difference in the mean MFM SIR on the surgical side among the three groups at follow-up (*p* = 0.039). The post hoc test showed that a significant difference was present between groups 1 and 3 (*p* < 0.001). The percentage of change for the SIR on the surgical side was the highest in group 3 (*p* < 0.001), with a significant difference among the three groups (*p* = 0.047). A significant difference was observed between groups 1 and 3 (*p* = 0.030). There was no significant difference between preoperative and follow-up MFM SIR on the contralateral side (Figure 3).

### 3.4. MFM CSA

The ipsilateral MFM CSA significantly decreased at the follow-up MRI in groups 2 (*p* = 0.04) and 3 (*p* = 0.006). However, the decrease in ipsilateral MFM CSA was not statistically significant in group 1. Mean MFM CSA on the postoperative MRI at the ipsilateral side was 728.27 ± 132.00 mm^2^ (−16.95 mm^2^ relative to baseline), 695.77 ± 101.52 mm^2^ (−66.77 mm^2^ relative to baseline), and 646.03 ± 118.67 mm^2^ (−110.17 mm^2^ relative to baseline) in groups 1, 2, and 3, respectively. Significant differences were found between the groups (*p* = 0.043). A significant difference was found between groups 1 and 3 (*p* = 0.025). The percentage of change for the MFM CSA on the surgical side significantly differed among the three groups (*p* = 0.022). Post hoc tests demonstrated that there was a significant difference between groups 1 and 3 (*p* < 0.001). On the contralateral side, postoperative changes in MFM CSA did not significantly differ compared to the preoperative values in any of the groups (Figure 3).

## 4. Discussion

The present study reported quantitative analyses of MFM injury measured by imaging of the MFM on MRI in patients with negative treatment outcomes after MLD. The results of this trial showed that changes in spine anatomy can occur after MLD. Significant MFM alterations were identified during postoperative MRI assessments, specifically on the surgical side, among patients who experienced unsatisfactory treatment outcomes following MLD. The MFM changes exhibited a distinct inclination towards an increase during follow-up after MLD, and significant changes in the MFM CSA and SIR of the MFM on the ipsilateral side were found compared to baseline MRI scans in group 2 and group 3. On the contralateral side, postoperative MFM CSA changes compared to the baseline did not significantly differ in any groups. No significant correlation was found between the surgery time and MFM changes, showing that operation time did not affect the MFM status.

MFM injury and atrophy have commonly been observed following traditional open approaches to the lumbar spine, and these changes have been associated with the development of LBP [30,31,32]. Previously published reports have highlighted the significance of minimizing muscle injury during surgical procedures, particularly in relation to postoperative low-back pain and its correlation with muscle denervation and atrophy [33,34]. Therefore, there has been a considerable amount of research and development of minimally invasive surgical techniques that aim to reduce the morbidity and iatrogenic muscle injury associated with traditional invasive spine procedures while achieving comparable outcomes [15,35]. MLD has become the preferred treatment for disc prolapse, known as the “gold standard,” due to its early patient recovery and rapid return to work [36,37]. In the present study, we analyzed the changes in the anatomy of the MFM in patients who experienced negative treatment outcomes regarding pain scores following primary single-level MLD, and we were able to quantify these changes at an average of 6 months after surgery. The MRI scans taken after performing MLD revealed significant changes in the MFM on the ipsilateral side. Previous studies have reported significant correlations between decreased CSA of the MFM and erector spinae muscles and increased LBP [38,39,40]. The results of this current study confirm the hypothesis that direct paraspinal muscle atrophy may be one of the most important causes of back pain after surgery. Recently, a systematic review including a total of six studies with 489 patients investigated the association between changes in MFM morphology and pain scores after discectomy surgery for LDH. In this review, a negative association was reported between a greater reduction in the CSA of the MFM and a lesser reduction in pain scores following discectomy surgery [41]. In this present study, although we found that MFM atrophy may be one of the most important causes of back pain after MLD, we did not investigate the changes in the MFM after MLD in patients with positive treatment outcomes. Moreover, we did not perform MRIs for the same patients consecutively over the three periods of time. While patients were selected based on inclusion and exclusion criteria, a large-scale, prospective, multi-center trial may provide homogeneity and answer the questions regarding the changes in the MFM over time.

In this present study, we divided the patient sample into three groups based on the interval between the surgery and the postoperative MRI evaluation and examined the relationship between the duration of follow-up and changes in the status of the MFM. A negative correlation was found between the follow-up period and the percentage of change for the surgical MFM CSA, showing that MFM volume significantly decreased as the follow-up period increased. The postoperative MFM CSA was significantly smaller than the preoperative MFM CSA at the surgical site in groups 2 and 3. However, the postoperative MFM CSA was greater than the preoperative MFM CSA at the surgical site, without a significant difference in group 1. Considering the mean follow-up time was approximately 11 days in group 1, this issue can be interpreted by the edema and inflammation in the early postoperative period. Prior research has documented the occurrence of muscle swelling resulting from edema for up to 10 months after surgery, highlighting the importance of conducting extended postoperative monitoring to identify these persistent alterations [25]. Furthermore, our results demonstrated an increase in signal intensity after MLD as the follow-up period progressed, aligning with previous studies that reported irreversible changes in paravertebral muscle signal intensity following posterior lumbar surgery [42,43].

The length of the surgical procedure plays a significant role in muscle injury, regardless of the specific surgical intervention being performed [44,45]. Theoretically, decreasing procedure time could minimize muscle injury. Previous research reported that the paraspinal muscle injury was correlated with the operation time during spinal surgery [17,35,46]. Moreover, Gejo et al. reported that an increase in T2 signal intensity on serial MRIs is correlated with surgery time [38]. However, the results of the current trial reported that there was no association between the surgery time and the percentage of change in MFM CSA. It is important to note that this study included only subjects with negative treatment outcomes regarding pain scores following MLD. Therefore, it is necessary to assess the correlation between the MFM changes found on MRI and the duration of surgery by comparing the patients with positive and negative treatment outcomes.

The MFM, located deep within the back, exhibits a unique structure consisting of multiple muscle bundles that extend along the grooves near the spinous process bilaterally. Positioned in close proximity to the innermost aspect of the spine, it possesses the largest area among the paravertebral muscles. The MFM is the only paraspinal muscle innervated by a single nerve, playing a critical role in providing spinal stability [47]. Chronic lumbar degenerative diseases and lumbar surgery can cause long-term damage to the MFM. Oxidative stress and inflammation are responsible for MFM injury and atrophy following posterior spinal surgery [42]. The process of atrophy and fatty replacement of the MFM appears to perpetuate a negative cycle, starting with pain in the spine, potentially originating from the intervertebral discs or zygapophyseal joints. This pain leads to reflex inhibition of the MFM, followed by further atrophy and fatty replacement of the muscle. It’s important to note that even if the LBP is alleviated, normal MFM function may not resume, and decreased MFM function can contribute to recurring LBP [48,49]. To prevent further atrophy of the MFM and progressive degeneration, patients with chronic LBP and those who have undergone lumbar surgery should engage in long-term rehabilitation exercises to strengthen the lumbar muscles.

This study has several limitations that need to be noted. First, the impact of the duration of follow-up after surgical procedures on the results is important. Since the analyses were conducted to assess the relation between follow-up MRI and MFM anatomy, it is uncertain whether the findings can accurately predict long-term outcomes. Tracking changes in the MFM could be more effectively achieved by observing the same patients over an extended period of time. As this study was designed retrospectively, patients with negative treatment outcomes underwent MRI evaluations at different time points when they visited our clinic after surgery. Further evaluation of postoperative MFM condition following MLD requires a prospective trial with serial MRI evaluations at predetermined periods. Secondly, this study did not routinely record changes in quality of life, medication consumption, functional improvement, or psychological status, which are also important indicators of treatment success. Therefore, the outcome was limited to changes in pain scores only. Thirdly, the creatine phosphokinase level, which is used to quantify muscle damage, was not measured. Finally, some subjects were excluded from this study due to missing data, which is a common issue in retrospective studies.

## 5. Conclusions

The postoperative MRI scans revealed significant MFM changes on the surgical side in patients with negative treatment outcomes after MLD. The change in CSA and SIR of the MFM showed a tendency to increase as the interval to the follow-up MRI scan increased. Challenges in the management of failed back surgery syndrome remain; a comprehensive initial assessment to identify treatable causes, such as MFM atrophy, could be an important step in answering questions about improving efficacy. Considering the results of this current study, less traumatic surgical procedures to reduce MFM atrophy and postoperative core strengthening and spinal rehabilitation in the early period following spinal surgery could improve treatment outcomes. Future randomized, prospective studies are needed to investigate the association between MFM changes and treatment outcomes after MLD and confirm or refute the findings of this study.

## Figures and Tables

**Table 1:** Baseline characteristics.

Variables	Value
Patients	79
Age	55.89 ± 9.22
Gender	
Female	29 (36.7%)
Male	50 (63.3%)
BMI	28 ± 6.14
Operation level	
L3–L4	16 (20.2%)
L4–L5	33 (41.7%)
L5–S1	30 (37.9%)

Data presented as mean ± standard deviation or n (%).

**Table 2:** Baseline characteristics stratified by group.

Variables	Group 1	Group 2	Group 3	*p*
Patients (n)	22	27	30	
Age	58.63 ± 5.94	53.25 ± 9.73	56.26 ± 10.29	0.121
Gender				0.87
Female	8	9	12	
Male	14	18	18	
BMI	27.18 ± 1.53	26.71 ± 1.68	27.42 ± 2.21	
Surgery level				0.45
L3–L4	3	8	5	
L4–L5	12	9	12	
L5–S1	7	10	13	

Data presented as mean ± standard deviation or n (%). BMI: Body mass index.

**Table 3:** Surgery data and follow-up of study scales stratified by group.

	Total	Group 1 (n = 22)	Group 2 (n = 27)	Group 3 (n = 30)	*p*
Follow-up point (days)	130.41 ± 58.13	11.64 ± 8.58	95.16 ± 44.25	284.43 ± 84.23	<0.001
Surgery time (minutes)	75.11 ± 22.8	72.56 ± 22.57	75.65 ± 25.52	77.12 ± 20.14	0.773
VAS score					
Baseline	7.05 ± 1.18	7.13 ± 1.20	7.14 ± 1.26	6.9 ± 1.12	0.69
Follow-up point	4.59 ± 1.21	4.22 ± 1.34	4.77 ± 1.31	4.65 ± 0.98	0.26

Data presented as mean ± standard deviation. VAS: visual analog scale.

## Data Availability

Present with the corresponding author and can be delivered upon reasonable request.

## References

[1] Clark S., Horton R. (2018). Low back pain: A major global challenge. Lancet.

[2] Kjaer P., Tunset A., Boyle E., Jensen T.S. (2016). Progression of lumbar disc herniations over an eight-year period in a group of adult Danes from the general population—A longitudinal MRI study using quantitative measures. BMC Musculoskelet. Disord..

[3] Zielinska N., Podgórski M., Haładaj R., Polguj M., Olewnik Ł. (2021). Risk Factors of Intervertebral Disc Pathology—A Point of View Formerly and Today—A Review. J. Clin. Med..

[4] Jin L., Yin Y., Chen W. (2021). Role of the Lumbosacral Transition Vertebra and Vertebral Lamina in the Pathogenesis of Lumbar Disc Herniation. Orthop. Surg..

[5] Foster N., Anema J., Cherkin D. (2018). Prevention and treatment of low back pain: Evidence, challenges, and promising directions. Lancet.

[6] Lee J., Choi K., Kang S. (2019). Nonsurgical Treatments for Patients with Radicular Pain from Lumbosacral Disc Herniation. Spine J..

[7] Atlas S.J., Keller R.B., Wu Y.A., Deyo R.A., Singer D.E. (2005). Longterm outcomes of surgical and nonsurgical management of sciatica secondary to a lumbar disc herniation: 10 year results from the Maine lumbar spine study. Spine (Phila Pa 1976).

[8] Kreiner D., Hwang S., Easa J. (2014). An Evidence-based Clinical Guideline for the Diagnosis and Treatment of Lumbar Disc Herniation with Radiculopathy. Spine J..

[9] Haines S., Jordan N., Boen J. (2002). Discectomy Strategies for Lumbar Disc Herniation: Results of the LAPDOG Trial. J. Clin. Neurosci..

[10] Thomé C., Barth M., Scharf J., Schmiedek P. (2005). Outcome after lumbar sequestrectomy compared with microdiscectomy: A prospective randomized study. J. Neurosurg. Spine.

[11] Silverplats K., Lind B., Zoëga B. (2010). Clinical Factors of Importance for Outcome After Lumbar Disc Herniation Surgery: Long-term Follow-up. Eur. Spine J..

[12] Virk S., Diwan A., Phillips F., Sandhu H., Khan S. (2017). What is the Rate of Revision Discectomies After Primary Discectomy on a National Scale?. Clin. Orthop..

[13] Freeman M., Woodham M., Woodham A. (2010). The Role of the Lumbar Multifidus in Chronic Low Back Pain: A Review. PM R.

[14] Wilke H., Wolf S., Claes L., Arand M., Wiesend A. (1995). Stability of the lumbar spine with different muscle groups. Spine.

[15] Fan S., Hu Z., Zhao F., Zhao X., Huang Y., Fang X. (2010). Multifidus muscle changes and clinical effects of one-level posterior lumbar interbody fusion: Minimally invasive procedure versus conventional open approach. Eur. Spine J..

[16] Tsutsumimoto T., Shimogata M., Ohta H., Misawa H. (2009). Mini-open Versus Conventional Open Posterior Lumbar Interbody Fusion for the Treatment of Lumbar Degenerative Spondylolisthesis: Comparison of Paraspinal Muscle Damage and Slip Reduction. Spine.

[17] Ahn J., Lee H., Park E. (2019). Multifidus Muscle Changes After Biportal Endoscopic Spinal Surgery: Magnetic Resonance Imaging Evaluation. World Neurosurg..

[18] Sadeghi S., Bible J.E., Cortes D.H. (2020). Quantifying Dysfunction of the Lumbar Multifidus Muscle After Radiofrequency Neurotomy and Fusion Surgery: A Preliminary Study. J. Eng. Sci. Med. Diagn. Ther..

[19] Smuck M., Crisostomo R., Demirjian R., Fitch D., Kennedy D., Geisser M. (2015). Morphologic Changes in the Lumbar Spine After Lumbar Medial Branch Radiofrequency Neurotomy: A Quantitative Radiological Study. Spine J..

[20] Pishnamaz M., Schemmann U., Herren C. (2018). Muscular Changes After Minimally Invasive Versus Open Spinal Stabilization of Thoracolumbar Fractures: A Literature Review. J. Musculoskelet. Neuronal Interact..

[21] Gold G. (2003). Dynamic and Functional Imaging of the Musculoskeletal System. Semin. Musculoskelet. Radiol..

[22] Kikuchi Y., Nakamura T., Takayama S., Horiuchi Y., Toyama Y. (2003). MR imaging in the diagnosis of denervated and reinnervated skeletal muscles: Experimental study in rats. Radiology.

[23] Gejo R., Kawaguchi Y., Kondoh T., Tabuchi E., Matsui H., Torii K., Ono T., Kimura T. (2000). Magnetic resonance imaging and histologic evidence of postoperative back muscle injury in rats. Spine.

[24] Storheim K., Berg L., Hellum C., Gjertsen Ø., Neckelmann G., Espeland A., Keller A., Norwegian Spine Study Group (2017). Fat in the lumbar multifidus muscles—Predictive value and change following disc prosthesis. surgery and multidisciplinary rehabilitation in patients with chronic low back pain and degenerative disc: 2-year follow-up of a randomized trial. BMC Musculoskelet. Disord..

[25] Faur C., Patrascu J., Haragus H., Anglitoiu B. (2019). Correlation between multifidus fatty atrophy and lumbar disc degeneration in low back pain. BMC Musculoskelet. Disord..

[26] Koebbe C.J., Maroon J.C., Abla A., El-Kadi H., Bost J. (2002). Lumbar microdiscectomy: A historical perspective and current technical considerations. Neurosurg. Focus..

[27] Dowling T.J., Munakomi S., Dowling T.J. (2023). Microdiscectomy. StatPearls [Internet].

[28] Tonomura H., Hatta Y., Mikami Y., Ikeda T., Harada T., Nagae M., Koike H., Hase H., Kubo T. (2017). Magnetic Resonance Imaging Evaluation of the Effects of Surgical Invasiveness on Paravertebral Muscles After Muscle-preserving Interlaminar Decompression (MILD). Clin. Spine Surg..

[29] Langley G.B., Sheppeard H. (1985). The visual analogue scale: Its use in pain measurement. Rheumatol. Int..

[30] Stevens K., Spenciner D., Griffiths K., Kim K., ZwienenbergLee M., Alamin T. (2006). Comparison of Minimally Invasive and Conventional Open Posterolateral Lumbar Fusion Using Magnetic Resonance Imaging and Retraction Pressure Studies. J. Spinal Disord. Tech..

[31] Suwa H., Hanakita J., Ohshita N., Gotoh K., Matsuoka N., Morizane A. (2000). Postoperative changes in paraspinal muscle thickness after various lumbar back surgery procedures. Neurol. Med. Chir..

[32] Taylor H., McGregor A., Medhi-Zadeh S., Richards S., Kahn N., Zadeh J. (2002). The Impact of Self-retaining Retractors on the Paraspinal Muscles During Posterior Spinal Surgery. Spine.

[33] Laasonen E. (1984). Atrophy of Sacrospinal Muscle Groups in Patients with Chronic, Diffusely Radiating Lumbar Back Pain. Neuroradiology.

[34] Sihvonen T., Herno A., Palja¨rvi L., Airaksinen O., Partanen J., Tapaninaho A. (1993). Local denervation atrophy of paraspinal muscles in postoperative failed back syndrome. Spine.

[35] Arts M., Brand R., van der Kallen B., Lycklama à Nijeholt G., Peul W. (2011). Does minimally invasive lumbar disc surgery result in less muscle injury than conventional surgery? A randomized controlled trial. Eur. Spine J..

[36] Ozalp H. (2018). Disk Hernilerinde Altın Standart: Mikrodiskektomi. Turk Norosirurji Dergsi.

[37] Kazemi N., Crew L.K., Tredway T.L. (2013). The future of spine surgery: New horizons in the treatment of spinal disorders. Surg. Neurol. Int..

[38] Beneck G., Kulig K. (2012). Multifidus atrophy is localized and bilateral in active persons with chronic unilateral low back pain. Arch. Phys. Med. Rehabil..

[39] Wallwork T., Stanton W., Freke M., Hides J. (2009). The Effect of Chronic Low Back Pain on Size and Contraction of the Lumbar Multifidus Muscle. Man Ther..

[40] He K., Head J., Mouchtouris N., Hines K., Shea P., Schmidt R., Hoelscher C., Stricsek G., Harrop J., Sharan A. (2020). The Implications of Paraspinal Muscle Atrophy in Low Back Pain, Thoracolumbar Pathology, and Clinical Outcomes After Spine Surgery: A Review of the Literature. Glob. Spine J..

[41] Lu H., Wang L., Li M., Chen X. (2022). The Association Between Changes in Multifidus Muscle Morphology and Back Pain Scores Following Discectomy Surgery for Lumbar Disc Herniation: A Systematic Review and Meta-analysis. Eur. Spine J..

[42] Gejo R., Matsui H., Kawaguchi Y., Ishihara H., Tsuji H. (1999). Serial changes in trunk muscle performance after posterior lumbar surgery. Spine.

[43] Gille O., Jolivet E., Dousset V., Degrise C., Obeid I., Vital J.M., Skalli W. (2007). Erector spinae muscle changes on magnetic resonance imaging following lumbar surgery through a posterior approach. Spine.

[44] Motosuneya T., Asazuma T., Tsuji T., Watanabe H., Nakayama Y., Nemoto K. (2006). Postoperative change of the cross-sectional area of back musculature after 5 surgical procedures as assessed by magnetic resonance imaging. J. Spinal Disord. Tech..

[45] Datta G., Gnanalingham K., Peterson D., Mendoza N., O’Neill K., Van Dellen J., McGregor A., Hughes S. (2004). Back pain and disability after lumbar laminectomy: Is there a relationship to muscle retraction?. Neurosurgery.

[46] Arts M., Nieborg A., Brand R., Peul W. (2007). Serum creatine phosphokinase as an indicator of muscle injury after various spinal and nonspinal surgical procedures. J. Neurosurg. Spine.

[47] Rosatelli A.L., Ravichandiran K., Agur A.M. (2008). Threedimensional study of the musculotendinous architecture of lumbar multifidus and its functional implications. Clin. Anat..

[48] Wang X., Jia R., Li J. (2021). Research Progress on the Mechanism of Lumbarmultifidus Injury and Degeneration. Oxid. Med. Cell. Longev..

[49] Shahidi B., Hubbard J., Gibbons M., Ruoss S., Zlomislic V., Allen R., Garfin S., Ward S. (2017). Lumbar multifidus muscle degenerates in individuals with chronic degenerative lumbar spine pathology. J. Orthop. Res..

